# Amlodipine decreases mitral regurgitation volume in dogs over 7 days: A study of 24 dogs with myxomatous mitral valve degeneration

**DOI:** 10.1002/vro2.33

**Published:** 2022-04-05

**Authors:** Sool Yi Park, Won‐Seok Oh, Seunggon Lee

**Affiliations:** ^1^ Seoul Animal Heart Hospital Gangnam‐gu Seoul Republic of Korea; ^2^ Dr. Oh's Hwanggum Geriatric Animal Medical Center Jung‐gu Daegu Republic of Korea

## Abstract

**Background:**

Amlodipine, a dihydropyridine calcium‐channel blocker, is currently being investigated as a treatment for myxomatous mitral valvular degeneration (MMVD). However, the effects of amlodipine on moderate or severe spontaneous MMVD, based on changes in echocardiographic indices, remain unclear.

**Animals:**

Client‐owned small‐breed dogs (*n* = 24) with naturally occurring MMVD of the American College of Veterinary Internal Medicine (ACVIM) stage B2 or higher.

**Methods:**

Basic dog information including previous medication treatments were recorded. All subjects received amlodipine 0.1 mg/kg, administered per os, twice daily for 7 days, in addition to their existing medication. We measured systolic blood pressure, obtained x‐ray, echocardiography, blood test data before and after 1 week of amlodipine administration.

**Results:**

Left ventricular end‐diastolic internal diameter, left atrial diameter and E wave reduced statistically after 1 week of amlodipine treatment (all *p* < 0.001). No adverse effects were reported.

**Conclusions:**

These findings suggest that low‐dose amlodipine should be considered as treatment for dogs with ACVIM stage B2‒C MMVD.

## INTRODUCTION

Myxomatous mitral valvular degeneration (MMVD) is common in small‐breed dogs and leads to heart failure (HF). Amlodipine, a dihydropyridine calcium‐channel blocker, is a well‐known first‐line antihypertensive treatment in dogs and cats.^1^ Amlodipine acts as an arteriolar vasodilator by acting on the voltage‐gated calcium channels of the heart and smooth muscle, decreasing systolic blood pressure (SBP) and reducing the afterload.^2^, ^3^ This also reduces left atrial pressure (LAP) and mitral valve regurgitation (MR).^4^, ^5^, ^6^ Amlodipine has been investigated for its therapeutic effects on MMVD. However, there have been very few prospective studies evaluating the effects of amlodipine on moderate or severe spontaneous MMVD. Moreover, there are currently few studies that have investigated the effects of amlodipine based on changes in echocardiographic indices, as described in the American College of Veterinary Internal Medicine (ACVIM) guidelines.^1^ This is a prospective case series investigating the short‐term effect of amlodipine on the echocardiographic indices in small‐breed dogs with naturally occurring MMVD and ACVIM stage B2 or higher.

## MATERIALS AND METHODS

### Study design

This was a prospective, non‐randomised, experimental study.

### Animals

Among the cases admitted to Seoul Animal Heart Hospital between 2019 and 2020, dogs diagnosed with ACVIM stage B2–C (subclinical case in compensatory phase after pulmonary oedema) MMVD, who had no severe systemic disease and stable underlying health, were enrolled in this study. Middle to large‐breed dogs were excluded and only small‐breed dogs (*n* = 24) were included, to include dogs of similar weight. We chose to include only dogs with a bodyweight of <9 kg. All cases were client‐owned dogs. The owners provided consent for participation in the trial, including the administration of amlodipine. We excluded all dogs with comorbidities. Subjects in this study were diagnosed with ACVIM stage ≥B2 MMVD and did not have any other comorbidities. For each dog, data on age, weight, sex, breed and previous medication treatments were obtained. We obtained systemic blood pressure (SBP), radiographs, vertebral heart score (VHS), echocardiography and blood test (blood urea nitrogen (BUN), creatinine, phosphorus) data at 1 week intervals, before and after the 7‐day period of treatment. These assessments were excluded in dogs that were not blood tested at the owner's request.

### Echocardiography

Echocardiography was performed using a 2.9/5.8‐MHz sector transducer (GE Vivid E95 4D echo machine with R2 software, GE Medical Systems, Chicago, IL, USA). The test consisted of 2D, M‐mode, spectral Doppler and colour flow Doppler echocardiography; the ACVIM grading guideline indices were measured.^1^ For left ventricular end‐diastolic diameter normalised for bodyweight (LVIDdN), left ventricular end‐diastolic internal diameter (LVIDD), left atrial diameter (LAD), aortic root diameter (AoD), LAD/AoD ratio, the values were obtained with either M‐mode or 2D image, in the right parasternal short‐axis view. The peak transmitral early diastolic wave (E wave) velocity and atrial contraction wave (A wave) were measured in the left parasternal apical four‐chamber view using Doppler echocardiography. The sample volume was measured at the mitral valve leaflet tip.^7^

### Medication

All dogs received amlodipine (Norvasc 5 mg tablet; Pfizer Pharmaceuticals Korea, Seoul, Korea) 0.1 mg/kg, administered per os (PO), twice daily by the owner for 7 days, in addition to their existing pharmacological therapy. The dogs were receiving pimobendan, furosemide, enalapril or spironolactone combination therapy, depending on their condition. All dogs were receiving pimobendan (0.2‒0.4 mg/kg PO twice daily). Enalapril (0.5 mg/kg PO twice daily), spironolactone (0.5‒1 mg/kg PO twice daily) or furosemide (0.5‒2.5 mg/kg PO twice daily) was co‐administered depending on their status. No other changes were made to the canines’ pharmacological treatments and they were maintained on the same diet.

### Statistical analyses

All non‐parametric tests were performed for each measured value regardless of the normality test results. For the variables presented in Table 1, frequency analysis and descriptive analysis were performed. For the variables presented in Table 2, Wilcoxon signed‐rank tests were performed. SPSS version 25.0 (IBM Inc., Armonk, NY, USA) was used for all statistical analyses. A *p*‐value <0.01 was considered statistically significant.

**TABLE 1:** Baseline characteristics of subjects (*n* = 24)

Variables	Classification	*n*	%	Median (interquartile range 25–75)
Age				13 (10.25–14.00)
Sex	Neutered male	10	41.7	
	Female	1	4.1	
	Spayed female	13	54.2	
Bodyweight				3.31 (2.71–4.55)
Breed	Chihuahua	1	4.2	
	Maltese	11	45.8	
	Mixed	5	20.8	
	Pomeranian	2	8.2	
	Schnauzer	1	4.2	
	Shih tzu	1	4.2	
	Spitz	1	4.2	
	Toy poodle	1	4.2	
	Yorkshire terrier	1	4.2	

**TABLE 2:** Changes after 1 week of amlodipine treatment (*n* = 24)

	Median (interquartile range)			
Variables	Before	After (week 1)	*z*	*p*
LVIDD (cm)	2.67 (2.50–2.95)	2.44 (2.25–2.86)	−3.872	<0.001
LVIDdN	1.87 (1.77–1.97)	1.72 (1.57–1.83)	−3.915	<0.001
LAD (mm)	24.28 (22.71–25.91)	23.21 (21.45–24.44)	−3.407	0.001
AoD (mm)	13.08 (11.58–13.90)	13.08 (11.58–13.90)	0.000	1.000
LA/Ao	1.96 (1.70–2.01)	1.77 (1.63–1.94)	−2.754	0.006
E wave (m/s)	1.29 (1.01–1.50)	1.10 (0.87–1.24)	−4.053	<0.001
A wave (m/s)	1.00 (0.90–1.28)	1.00 (0.72–1.10)	−1.797	0.072
VHS (*n* = 4)	11.35 (11.05–11.50)	11.35 (10.68–11.50)	−1.000	0.317
SBP (mmHg) (*n* = 16)	140.00 (130.00–140.00)	130.00 (120.00–140.00)	−0.240	0.811
BUN (mg/dl) (*n* = 22)	19.80 (13.67–27.30)	20.90 (13.38–27.78)	−0.211	0.833
Creatinine (mg/dl) (*n* = 22)	0.68 (0.59–0.97)	0.69 (0.61–0.82)	−1.712	0.087
Phosphorus (mg/dl) (*n* = 7)	4.40 (3.90–4.70)	3.90 (3.20–4.00)	−2.201	0.028

*Note*: *z*—Wilcoxon signed‐rank test.

Abbreviations: AoD, aortic root diameter; BUN, blood urea nitrogen; LAD, left atrial diameter; LVIDD, left ventricular end‐diastolic internal diameter; LVIDdN, left ventricular end‐diastolic diameter normalised for bodyweight; SBP, systolic blood pressure; VHS, vertebral heart score.

## RESULTS

Significant changes in LVIDD, LVIDdN, LAD and E wave were observed before and after amlodipine treatment (all *p* < 0.001). LVIDdN, which forms part of the ACVIM MMVD grading criteria, with LVIDD, were decreased by 8% (2.2 mm). LAD and consequently the LA/Ao ratio, was decreased by 6.4% (1.57 mm) (Table 2). The E wave was decreased by 14% (0.18 m/s) (Table 2). During the 7‐day administration period no adverse effects were reported. No gingival hyperplasia or significant decrease in blood pressure or increase in renal function indicators (BUN, creatinine and phosphorus) were observed in blood tests performed after 1 week of amlodipine administration. There were no evident changes in the animals’ behaviour such as reduced appetite or lethargy.

## DISCUSSION

The results showed that the administration of low‐dose amlodipine (amlodipine 0.1 mg/kg PO twice daily) to client‐owned small‐breed dogs with spontaneous MMVD (ACVIM B2–C) led to a decrease in LVIDD, E flow and LA/Ao. In addition, no abnormal decrease in SBP or increase in the renal function panel (BUN, creatinine and phosphorus) was observed after the drug administration.

Previous studies have reported positive effects of amlodipine, including a significant decrease in LAP, in dogs with experimentally induced MMVD;^6^, ^8^ also in dogs with congestive HF caused by mitral valve disease and treated with multiple drugs after amlodipine administration.^9^ However, these studies were mostly conducted in laboratory animals;^6^, ^8^ or as a retrospective study.^9^ In addition, most studies were not conducted in dogs with spontaneous MMVD with ACVIM stage B2 or higher and that are considered to require the use of amlodipine in clinical practice. Dogs in those studies either had experimentally induced MMVD or were healthy dogs. In contrast, our prospective study was conducted in dogs with naturally occurring ACVIM stage B2‒C MMVD.

For the diagnosis and treatment of MMVD an indirect assessment of LAP is crucial.^10^ The echocardiography indicators LVIDdN, LVID, E flow and LA/Ao are central to the classification of MMVD in the ACIVM guidelines^11^ because these indicators are associated with changes in LAP and LA filling pressure.^12^, ^13^, ^14^, ^15^, ^16^ In this study, LA/Ao, LVIDD, LVIDdN and E wave were all reduced after amlodipine administration. Although a direct measurement of the LAP could not be performed, as previously reported,^6^ the improvement in echocardiography indicators suggests an association with the improvement of LAP and LA filling pressure.

The target subjects for amlodipine administration in this study are debatable because there is no guideline regarding the optimal timing for starting amlodipine in dogs with MMVD.^11^ Previous studies that evaluated the effects of amlodipine did not provide a clear standard in terms of initiating amlodipine therapy and the appropriate population,^9^ because it was administered in MMVD cases that had surgically induced MR,^6^ or the studies had a retrospective design with irregular timing. Consequently, further studies are needed to determine the optimal timing for starting amlodipine in dogs with MMVD.

No guidelines have been developed regarding the optimal amlodipine dose in dogs with MMVD. One recommended dose is 0.1‒0.5 mg/kg once daily in dogs with systemic arterial hypertension.^17^ In another study on the effect of amlodipine (0.2 mg/kg PO twice daily) on MMVD in dogs with experimentally induced MR, a decrease in the LAP was observed.^6^ In another study assessing the effect of a multidrug treatment, including amlodipine, on MMVD cases with HF, a dose of 0.25 mg/kg/day was associated with prolonged survival time.^9^ In a study in which 0.2 mg/kg PO twice daily of amlodipine was administered, a significant decrease in SBP was observed.^6^ In our study, the estimated LAP was decreased in association with the administration of amlodipine although the dose was half (0.1 mg/kg PO twice daily) that used in the other study; the reduced estimated LAP was not associated with a significant drop in blood pressure or an increase in the renal function panel (BUN, creatinine and phosphorus).

Th dose we used may not be optimal and it is premature to suggest that amlodipine could be considered as a treatment option based on the current study. However, it is noteworthy that the dose used was effective in that 33% of MMVD dogs, who were ACVIM B2 or greater (16/24 of them had LA:Ao 1.9), showed reduced effects in estimated LAP, LVIDD, LA/Ao and no adverse effects were reported. Originally, a dose of 0.2 mg/kg once daily was to be used; however, given that the dogs were already receiving other heart medications with twice daily dosing, the twice daily dose of amlodipine was applied to ease the drug preparation and administration for the owners.

We found no change in renal indices after 1 week of amlodipine administration. The effect of amlodipine on the glomerular filtration rate (GFR) varies according to the dose and the animal's health.^1^, ^18^ After administration of a high dose (0.57 mg/kg twice daily PO), a notable increase in BUN was reported.^24^ In humans, a change in the GFR according to the amlodipine dose and renal condition has been reported;^20^, ^21^, ^22^ however, studies regarding effects on renal indices are lacking in animals. The amlodipine dose that is associated with minimum impact on the GFR in dogs remains unknown. Even so, our study was not associated with changes in renal function.

There were limitations of this study. Firstly, the small sample size of 24 dogs. This posed a risk of an over‐ or underestimation during statistical analyses. It was challenging to recruit potential cases because the criteria for amlodipine administration was ACVIM stage B2 or C with an increased risk of HF during the administration of a heart medication. We monitored the therapeutic response after 1 week of amlodipine administration as in a previous study.^6^ A longer period of repeated measurements may have increased the reliability of the test results.

We could not identify an optimal dosage of amlodipine to reduce LAP, without apparent side effects, in this study. It is noteworthy that compared to a previous study, in which 0.2 mg/kg PO twice daily amlodipine showed apparent reduction in LAP,^6^ in our study, at half that dose, some 33% of the dogs showed an apparent improvement in LAP. A longer term study comprising a prospective randomised controlled trial based on the 0.1 mg/kg twice daily dose, may confirm that the positive effect on LAP is maintained.

The dogs in this study were already receiving furosemide, enalapril or pimobendane, with amlodipine added. Furosemide and enalapril are known to have an impact on systemic arterial pressure,^1^, ^19^, ^23^, ^24^ while enalapril may show an interaction with amlodipine with combined administration.^19^ As the dogs received varied drugs, the consequent interactions might have exerted an influence on the results of this study.

## CONFLICT OF INTEREST

The authors declare they have no conflicts of interest.

## FUNDING INFORMATION

The authors received no specific funding for this work.

## ETHICS STATEMENT

All dogs are owned by clients. All clients agreed to use data from their dogs.

## Data Availability

The data that supports the findings of this study are available in the supplementary material of this article.
